# Nanoclay, calcium alginate, and composite soil amendments mitigate drought effects on wheat growth and water-use efficiency

**DOI:** 10.3389/fpls.2026.1846027

**Published:** 2026-07-15

**Authors:** Ali El-Keblawy, Abdelaziz Elgamouz, Mohamed Abdallah, Mohamed G. Arab, Khalifa M. Alteneiji, Razan F. Alhammadi, Lamya Alsuwaidi, Walid Abo Baker, Mohammad A. Al-Faqieh, Soumya Koippully Manikandan

**Affiliations:** 1 Department of Applied Biology, University of Sharjah, Sharjah, United Arab Emirates; 2 Advanced Biotechnology Center, Research Institute of Sciences and Engineering, University of Sharjah, Sharjah, United Arab Emirates; 3 Faculty of Pharmacy, Alsalam University, Tanta, Egypt; 4 Department of Chemistry, College of Science, University of Sharjah, Sharjah, United Arab Emirates; 5 Department of Civil and Environmental Engineering, University of Sharjah, Sharjah, United Arab Emirates; 6 Agriculture and Livestock Department, Sharjah Government, Sharjah, United Arab Emirates

**Keywords:** arid agriculture, canopy dynamics, deficit irrigation, gas exchange, nanoclay, water-use efficiency

## Abstract

**Introduction:**

Water scarcity and increasing drought frequency constrain wheat production in arid and semi-arid regions, highlighting the need for soil-based strategies that can sustain crop performance under limited water availability.

**Methods:**

This study evaluated the effects of nanoclay (CN), calcium alginate (CG), and CN–CG composite formulations on wheat (*Triticum aestivum* L.) grown under greenhouse conditions. Plants were maintained at 60% water-holding capacity (WHC) under well-watered conditions or 20% WHC under drought. Growth traits, canopy proxy traits, leaf gas exchange, chlorophyll fluorescence, and spectral indices were measured to assess integrated plant responses.

**Results:**

Drought markedly reduced shoot fresh weight, canopy proxy traits, and net CO_2_ assimilation in the untreated control. Under drought conditions, CN (0.5%) showed the strongest buffering effect on canopy development and growth, whereas CNG 31 (0.5%) and CG (0.5%) maintained the highest net CO_2_ assimilation and instantaneous water-use efficiency relative to the stressed control. Photochemical stress indicators showed weaker treatment-related variation than gas-exchange traits, indicating that the amendments reduced the severity of drought responses but did not fully prevent physiological stress. Type II ANOVA confirmed significant effects of water regime, treatment group, and their interaction on growth traits (P ≤ 0.01), indicating that amendment effects were most pronounced under drought.

**Conclusion:**

Nanoclay, calcium alginate, and composite formulations attenuated short-term vegetative and physiological drought responses in greenhouse-grown wheat. These findings identify promising amendment candidates for further validation under field conditions, including root traits, dry biomass, grain yield, seasonal water productivity, amendment persistence, and cost-benefit assessment.

## Graphical Abstract

**Figure:**
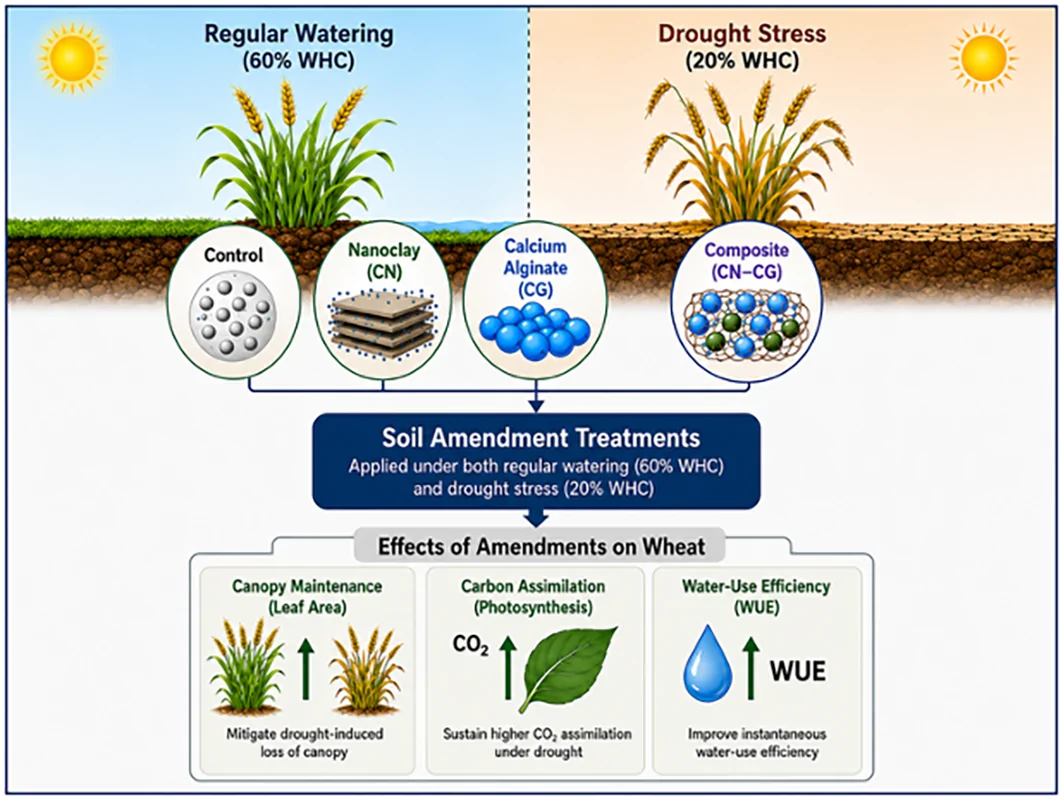

## Highlights

• Nanoclay and calcium alginate mitigated drought effects on wheat growth and physiology
• Canopy maintenance was the most sensitive indicator of drought mitigation
• Amendments partially maintained carbon gain and instantaneous WUE under drought
• Composite formulations integrated hydrogel and mineral benefits under drought

## 1 Introduction

Water scarcity and recurrent droughts are increasingly severe constraints on global wheat production, especially under climate change (Farooq et al., 2023; Elsayed et al., 2025). Arid and semi-arid regions, which account for a large share of rainfed wheat cultivation, face persistent water scarcity that critically restricts crop productivity (Nyaupane et al., 2024). Wheat (*Triticum aestivum* L.) is one of the world’s most important staple crops, providing approximately one-fifth of global dietary energy and protein (Saini et al., 2022). However, wheat is highly sensitive to water deficits, with drought stress frequently inducing early leaf senescence, reduced growth, and substantial yield losses that may exceed 50% under severe conditions (Nyaupane et al., 2024; Takács et al., 2025). Climate-driven increases in drought frequency and intensity therefore threaten both the productivity and stability of wheat-based food systems. Accordingly, improving wheat performance under water-limited conditions requires strategies that enhance water productivity rather than maximizing yield under optimal irrigation. Emphasis is increasingly placed on producing more biomass per unit of water supplied, particularly in arid and semi-arid systems where irrigation resources are constrained and deficit irrigation is widely practiced.

One promising approach to mitigating drought effects on crop growth and physiology is the use of soil amendments as water-management tools. Soil amendments, including organic conditioners, mineral additives, and polymer-based materials, can improve the soil’s moisture-retention capacity and thereby mitigate drought stress on plants (Chaves et al., 2009; Saha et al., 2020). Unlike genetic or breeding-based approaches that target plant traits directly, soil amendments operate by modifying the root-zone environment, improving water availability during periods of deficit. By enhancing soil structure, increasing water-holding capacity, and stabilizing nutrient availability, amendments can create a more resilient soil system that supports plant growth under reduced irrigation (Li et al., 2018; Abdallah et al., 2021). For example, superabsorbent hydrogels can absorb and store large quantities of water and release it gradually to the root zone, thereby reducing evaporative losses and increasing water-use efficiency (Saha et al., 2020; Malik et al., 2022). Experimental studies have shown that hydrogel-amended soils maintain higher moisture content, require less frequent irrigation, and support improved plant growth under drought conditions (Chang et al., 2021; Agbna and Zaidi, 2025). Similarly, clay-based amendments and other soil conditioners can enhance aggregation, reduce runoff, and improve infiltration, leading to greater plant-available water in drought-prone soils (Abd-Elsalam et al., 2024). Emphasizing low-input, sustainable soil amendments aligns with the growing need for environmentally sound strategies to mitigate agricultural water stress. Collectively, these approaches highlight soil management as a viable pathway for improving crop performance under drought conditions.

Among emerging soil amendment strategies, biopolymer-based hydrogels and mineral nanomaterials have attracted increasing attention due to their distinct and potentially complementary properties (Klein and Poverenov, 2020; Malik et al., 2022; Shanmugavel et al., 2024). Calcium alginate and nanoclay represent two such materials. Calcium alginate (CG) is a naturally derived biopolymer formed through ionic crosslinking of alginate chains with calcium ions, resulting in a hydrogel with a high capacity for water absorption and retention. Calcium alginate can function as a localized water reservoir, buffering plants against short-term water deficits (Hurtado et al., 2022; Shanmugavel et al., 2024). A recent study by Gomaa and Aldaby (2023) has demonstrated that alginate-based hydrogels significantly improve soil moisture availability and plant growth under drought, while also reducing nutrient leaching losses. However, the effectiveness of alginate-based amendments may be constrained by limited mechanical stability and biodegradability in soil (Dorochesi et al., 2023). In addition, interactions between alginate and soil nutrients can influence nutrient availability in complex ways that require further investigation. Thus, while calcium alginate offers clear benefits for water retention, its performance may be enhanced when combined with complementary materials.

Nanoclay (CN), in contrast, is a mineral-based amendment characterized by extremely fine clay particles with high surface area and strong surface charge properties (Nadziakiewicza et al., 2019). Nanoclays can improve soil physical properties, enhance water retention, and increase cation exchange capacity, thereby improving both moisture and nutrient availability (Das et al., 2022; Abd-Elsalam et al., 2024). Applications of nanoclay, including liquid nanoclay formulations, have been shown to substantially increase the water-holding capacity of sandy and degraded soils by binding soil particles and reducing percolation losses. These effects can improve seedling establishment, reduce irrigation frequency, and enhance plant growth under drought conditions (Abd-Elsalam et al., 2024). Nanoclays also improve nutrient retention by adsorbing essential ions, thereby reducing leaching and enhancing nutrient availability over time (Manjaiah et al., 2019; Fabiyi, 2023). Despite these advantages, nanoclay amendments face challenges with dispersion and aggregation. Without appropriate formulation, nanoclay particles may agglomerate, reducing their effective surface area and diminishing their functional benefits (Uddin et al., 2024; Gupta et al., 2025). Moreover, nanoclay performance varies with soil texture and environmental conditions, with more pronounced benefits often observed in coarse-textured soils. These considerations highlight the need for strategies that improve the stability and consistency of nanoclay performance in soil systems.

The contrasting strengths and limitations of calcium alginate and nanoclay suggest that composite soil amendments that combine biopolymer and mineral components may deliver integrated benefits. In such systems, nanoclay particles can reinforce the polymer matrix, improving the mechanical stability and durability of hydrogels, while the biopolymer component can enhance nanoclay dispersion and water retention. Experimental studies on polymer–clay composites have reported improved water-holding capacity, more gradual water release, and enhanced nutrient retention compared with single-component materials (Sarkar et al., 2014; Kong et al., 2019). These findings indicate that composite amendments may sustain soil moisture and nutrient availability more effectively under prolonged drought. Nevertheless, empirical evidence remains limited regarding optimal polymer–clay ratios, application rates, and crop-specific responses. Moreover, most studies have focused primarily on growth or biomass outcomes, with relatively little attention paid to the physiological mechanisms underlying improved drought performance.

Drought stress limits plant performance through coordinated effects on canopy development, gas exchange, and water-use efficiency. Reduced functional leaf area limits carbon assimilation, while changes in stomatal regulation alter the balance between carbon gain and water loss (Zhao et al., 2020; Fox et al., 2022; Nguyen et al., 2023). Integrative physiological traits, such as net CO_2_ assimilation rate and water-use efficiency, therefore provide critical insight into how soil amendments influence plant responses to water limitation. However, studies that explicitly link soil amendment effects to canopy-level and physiological processes remain scarce, particularly for composite formulations. In this context, several knowledge gaps persist. Comparative evaluations of nanoclay, calcium alginate, and their composite formulations under controlled drought conditions are limited, particularly across different application rates. In addition, few studies integrate growth, canopy traits, and gas-exchange physiology to assess how these amendments influence water-use performance rather than stress tolerance alone. Addressing these gaps is essential for developing soil-based strategies that improve crop performance under water-limited conditions.

The present study addresses these limitations by comparatively evaluating nanoclay (CN), calcium alginate (CG), and CN–CG composite formulations in wheat under well-watered and drought conditions. The study specifically examines how amendment formulation and rate influence vegetative growth-related traits, canopy proxy traits, gas-exchange parameters, instantaneous water-use efficiency, chlorophyll fluorescence, and reflectance-based indicators. In addition, cumulative recorded water addition required to restore pots to target WHC was quantified to support transparent interpretation of the water-addition responses. We hypothesized that composite nanoclay–alginate amendments would better maintain functional canopy area, carbon assimilation, and instantaneous water-use efficiency under drought than individual amendments, and that growth-related responses would be closely linked to physiological performance. The study is intended as a controlled greenhouse physiological assessment rather than a direct test of field-scale yield or agronomic water productivity.

## 2 Materials and methods

### 2.1 Plant material, experimental design, and growth conditions

Wheat (*Triticum aestivum* L., cv. Yecora Rojo) seeds were sown in plastic pots. After emergence, seedlings were thinned to five uniform plants per pot to ensure consistent plant density. The experiment was conducted under greenhouse conditions at the University of Sharjah, with natural photoperiod and day/night temperatures maintained at 22–26 °C. The study followed a completely randomized two-factor design with water regime and treatment group as fixed factors, using five replicate pots per treatment-water combination. The pot was treated as the experimental unit. Treatment group was defined as the untreated control or each amendment formulation-rate combination. Because application rate was not fully orthogonal across all amendment types, and the untreated control had no amendment rate, dose was incorporated within the treatment-group factor rather than analyzed as a separate independent factor.

### 2.2 Preparation of nanoclay, calcium alginate, and composite formulations

CN was prepared using a green synthesis approach based on locally sourced clay and date palm pit extract as a natural stabilizing and dispersing agent. Clay suspensions were subjected to controlled ultrasonication, followed by washing and drying to obtain nanoscale clay particles (Sarkar et al., 2014; Abd-Elsalam et al., 2024). CG was synthesized by ionic gelation of sodium alginate derived from brown seaweed. A 1% (w/v) sodium alginate solution was added dropwise to a 2% (w/v) calcium chloride solution under continuous stirring to form calcium alginate beads (Agulhon et al., 2012). The beads were thoroughly washed, oven-dried, and ground into fine particles prior to soil application. Nanoclay–alginate composite formulations (CNG) were prepared by combining CN and CG at defined mass ratios, followed by Ca²^+^ crosslinking using the same ionic gelation approach. Hydrodynamic particle size and polydispersity index (PDI) of CN, CG and composite suspensions were measured by dynamic light scattering (DLS) using a Zetasizer Advance Series (Ultra) (Malvern Panalytical, UK).

### 2.3 Soil amendment application and watering regimes

Soil amendments (CN, CG, or CNG formulations) were thoroughly mixed with soil at the designated rates prior to sowing. Each pot contained 230 g of air-dried soil (dry mass basis); therefore, amendment rates of 0.5% and 1.5% (w/w) corresponded to 1.15 g and 3.45 g total amendment per pot, respectively. For CG-only treatments, 0.25% and 0.5% (w/w) were used (i.e., 0.575 g and 1.15 g per pot). The amendment rates were selected as low and high screening levels for controlled greenhouse evaluation, based on preliminary formulation handling and the range commonly used in polymer–clay soil amendment studies. These rates were intended for physiological screening under pot conditions and should not be interpreted as field-rate recommendations. For composite treatments, the total amendment mass per pot was partitioned into CN and CG according to the specified CN: CG mass ratios: CNG 31 = 3:1 CN: CG, CNG 11 = 1:1 CN: CG, and CNG 13 = 1:3 CN: CG. These ratios are also summarized in **Supplementary Table 1**.

Two irrigation regimes were applied: well-watered conditions (R), with soil moisture maintained at 60% water-holding capacity (WHC), and drought stress (S), with soil moisture maintained at 20% WHC. Soil WHC was determined gravimetrically. For the 230 g soil mass used per pot, 100% WHC corresponded to 97.5 g water pot^-1^. Accordingly, the target water contents were 58.5 g water pot^-1^ under 60% WHC and 19.5 g water pot^-1^ under 20% WHC. Target pot mass was calculated as the initial dry pot mass plus the corresponding target water content. Pots were weighed at recorded intervals, and water was added to restore each pot to its assigned target mass. At each recorded weighing date, water addition was calculated as the positive difference between target pot mass and measured pot mass. The cumulative recorded water-addition variable therefore represents the amount of water required to restore pots to the assigned WHC target across recorded weighing dates, not a direct partitioned estimate of plant transpiration, evaporation, total evapotranspiration, or agronomic water productivity. To avoid nutrient limitation, all treatments received identical nutrient inputs using Hoagland’s nutrient solution (Hoagland and Arnon, 1950) and a balanced NPK fertilizer.

### 2.4 Growth measurements and leaf area estimation

Plants were harvested 60 days after sowing. Shoot length (cm), shoot fresh weight (g plant^-1^), and the number of green and yellow leaves were recorded. Shoot fresh weight was calculated as total shoot fresh mass per pot divided by the number of surviving plants. Dry biomass, root biomass, total plant dry biomass, and root-to-shoot ratio were not measured; therefore, shoot fresh weight was interpreted as a short-term vegetative growth indicator rather than as a substitute for dry biomass or yield.

Leaf area was estimated using the youngest fully expanded leaf from each plant, following established linear measurement approaches for wheat leaves (Chanda and Singh, 2002). Leaf length and maximum width were measured, and leaf area was calculated as 0.75 × length × width, where the correction factor accounts for the characteristic geometry of wheat leaves. The estimated green leaf area per plant was calculated by multiplying the estimated single-leaf area by the number of green leaves. It was used as a relative proxy for canopy development rather than an absolute leaf area index (Lopes and Reynolds, 2012).

### 2.5 Gas exchange and water-use efficiency

Leaf gas exchange was measured on fully expanded, intact green leaves using a portable photosynthesis system (CIRAS-4) during mid-morning to minimize diurnal variation. Measurements were conducted on 3–4 leaves per pot using the same leaf-position criterion across treatments; when more than one leaf was measured per pot, leaf-level values were averaged to obtain one pot-level replicate. Leaves were selected from the youngest fully expanded, non-senescent leaf position whenever possible. During measurements, chamber conditions were maintained at a photosynthetically active radiation (PAR) of 1000 µmol photons m^-2^ s^-1^, reference CO_2_ concentration of 400 µmol mol^-1^, relative humidity of 60%, vapor pressure deficit (VPD) of 1.5 kPa, leaf temperature of 25 °C, and enclosed leaf area of 0.5 cm². Net CO_2_ assimilation rate (A), stomatal conductance (g_s_), and transpiration rate (E) were recorded after readings stabilized. Instantaneous water-use efficiency (WUE) was calculated as A/E, reflecting the balance between carbon gain and transpirational water loss (Farquhar et al., 1989). This metric represents instantaneous leaf-level water-use efficiency rather than seasonal water use, plant water consumption, agronomic water productivity, or yield per unit water supplied.

### 2.6 Chlorophyll fluorescence, spectral indices, and chlorophyll estimation

Chlorophyll fluorescence was measured using a pulse-modulated fluorometer (FMS-2, Hansatech Instruments Ltd., Norfolk, UK) (Maxwell and Johnson, 2000; Hussain and Reigosa, 2021). The measurement configuration and parameter calculations followed Hussain and Reigosa (2021). Prior to measurement, plants were dark-adapted for 20 min, and leaves were clipped using dark-adaptation leaf clips. Fluorescence was then induced using a saturating pulse protocol, in which actinic irradiance was increased stepwise from 0 to 2000 µmol m^-2^ s^-1^, with fluorescence signals recorded at 10-s intervals. Variable fluorescence (F_v_) and maximum fluorescence (F_m_) were obtained, and the maximum photochemical efficiency of PSII (F_v_/F_m_) and the effective quantum yield of PSII (ΦPSII) were calculated accordingly.

Leaf spectral indices were measured non-destructively using a CI-710s SpectraVue Leaf Spectrometer (CID Bio-Science, USA) (Krasavtseva and Makarov, 2024). The instrument records leaf reflectance spectra, and vegetation indices were calculated using the manufacturer’s algorithms. The spectrometer was calibrated prior to measurements. For each plant, three fully expanded, intact leaves were assessed; each leaf was positioned in the leaf clip and measured three times at independent points to account for within-leaf variability. The following indices were derived from reflectance spectra: normalized difference vegetation index (NDVI), water band index (WBI), carotenoid reflectance index 1 (CRI1), and structure insensitive pigment index (SIPI). Total chlorophyll was estimated from the same reflectance spectra using the instrument’s chlorophyll estimation procedure. Chlorophyll values are reported as reflectance-derived estimates and were interpreted as indicators of relative pigment status rather than chemically determined chlorophyll concentrations.

### 2.7 Statistical analysis

All data were analyzed using linear models with water regime, treatment group, and their interaction as fixed effects. The model structure was response = water regime + treatment group + water regime × treatment group + error, with pot-level replicate values used as the observational units. Growth and physiological traits were evaluated using Type II analysis of variance (ANOVA), which is appropriate for factorial experimental designs (Montgomery, 2017). Means and 95% confidence intervals presented in figures and tables were derived from the fitted models. Percentage changes were calculated to quantify drought-induced effects within treatments and relative performance under drought compared with the stressed control. Statistical significance was assessed at P < 0.05. Figures were generated using Python (version 3.12.13).

Cumulative recorded water addition was analyzed using the same water regime × treatment group linear model. For each pot, cumulative recorded water addition was calculated as the sum of water added across recorded weighing dates to restore the pot to its assigned target WHC. Treatment-level reductions in recorded water addition were calculated relative to the corresponding untreated control within the same water regime using the following equation:


$$\text{Water-addition }{\text{reduction}}_{\text{i,r}}\;(\%)\;=\;[1\;-\;({\bar{\text{W}}}_{\text{i,r}}/{\bar{\text{W}}}_{\text{o,r}})]\times 100$$

where ${\bar{\text{W}}}_{\text{i,r}}$ is the mean cumulative recorded water addition for amendment treatment i under water regime r, and ${\bar{\text{W}}}_{\text{o,r}}$ is the mean cumulative recorded water addition for the untreated control under the same water regime. When ${\bar{\text{W}}}_{\text{i,r}}$ exceeded ${\bar{\text{W}}}_{\text{o,r}}$, the treatment was interpreted as showing no reduction relative to the control. Because evaporation and transpiration were not partitioned, these values were interpreted as water added to restore target WHC rather than as direct plant water consumption, total evapotranspiration, or whole-season water productivity.

## 3 Results

### 3.1 Growth responses of wheat to nanoclay, calcium alginate and composite amendments under drought

Hydrodynamic particle size characterization by DLS indicated sub-micron diameters for the prepared materials (CN: 500 nm; CG: 841 nm; composites: 548–714 nm) with polydispersity indices ranging from 0.2112 to 0.6325 (**Supplementary Table 2**). To evaluate amendment-mediated drought buffering at the vegetative stage, shoot fresh weight, shoot length, and estimated green leaf area per plant (canopy proxy) were quantified as vegetative growth-related traits. **Table 1** shows a pronounced drought-induced decline in the untreated control, whereas several amendments moderated this reduction to varying extents. In the control, drought decreased shoot fresh weight by 35.5% relative to well-watered condition (**Supplementary Table 3**), indicating a marked reduction in shoot fresh mass under water deficit. Amendment effects were most evident under drought and were formulation- and dose-dependent. The greatest mitigation was observed for the lower-dose single-component treatments: CN (0.5%) restricted the drought-associated reduction to 12.0%, while CG (0.5%) restricted it to 12.7%. Under drought, both treatments maintained markedly higher shoot fresh weight than the stressed control (**Table 1**). Composite treatments also improved shoot fresh weight under drought relative to the stressed control, although the magnitude of the shoot fresh-weight response varied among ratios and doses.

**Table 1:** Effects of nanoclay, calcium alginate, and their combinations on shoot fresh weight (means ± 95% confidence intervals) of wheat under well-watered and drought conditions.

Treatment	Well-watered	Drought
Control	0.73 ± 0.026	0.47 ± 0.030
CN (0.5%)	2.16 ± 0.100	1.90 ± 0.178
CN (1.5%)	2.60 ± 0.141	1.59 ± 0.184
CG (0.25%)	1.48 ± 0.212	0.93 ± 0.061
CG (0.5%)	1.81± 0.067	1.58 ± 0.064
CNG 31 (0.5%)	3.08 ± 0.386	1.83 ± 0.071
CNG 31 (1.5%)	3.47 ± 0.211	2.28 ± 0.251
CNG 11 (0.5%)	2.28 ± 0.051	1.69 ± 0.423
CNG 11 (1.5%)	2.47 ± 0.111	1.84 ± 0.179
CNG 13 (0.5%)	2.32 ± 0.212	1.87 ± 0.173
CNG 13 (1.5%)	2.71 ± 0.122	2.09 ± 0.130

**Figure 1:**
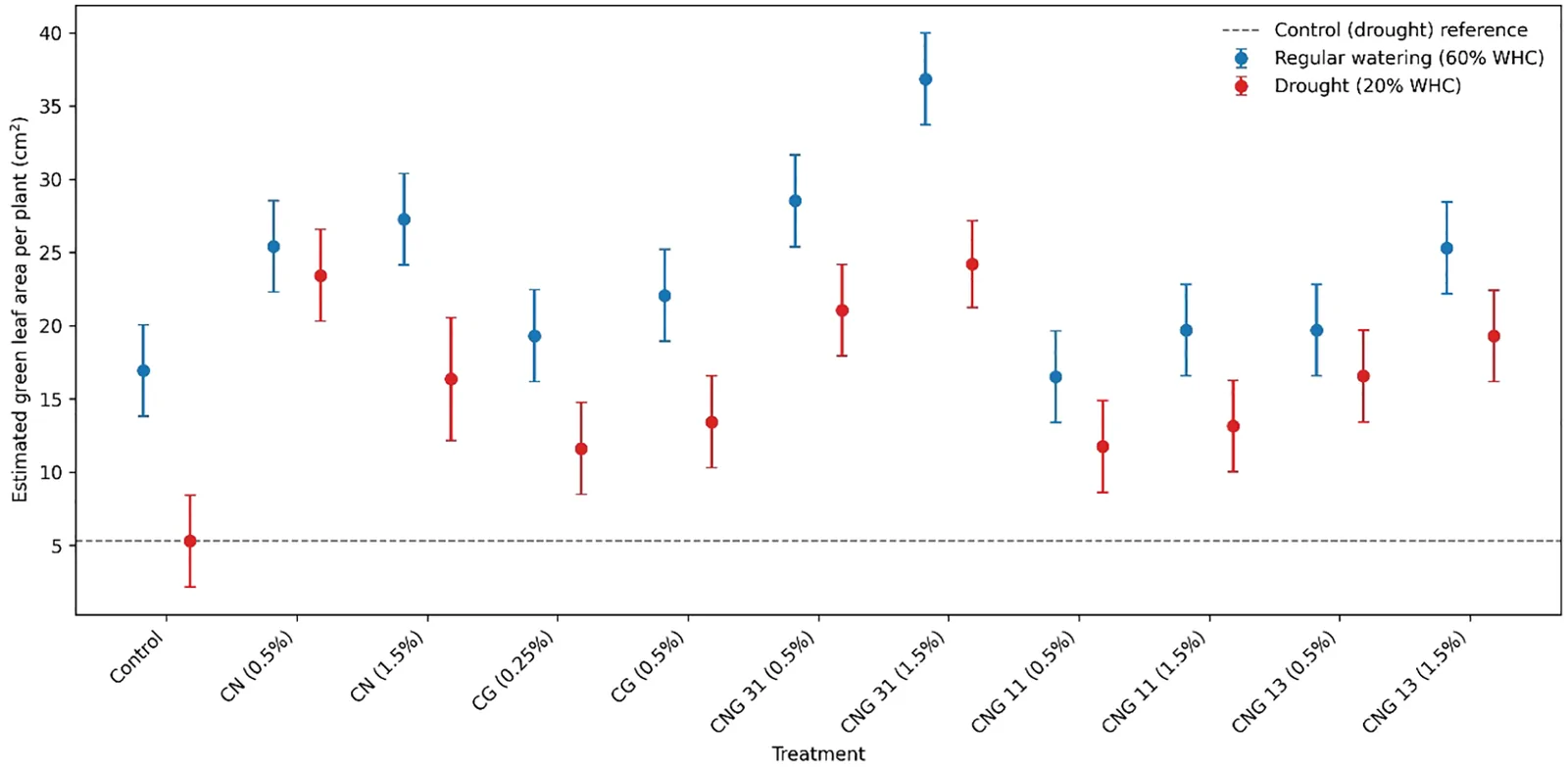
Effects of nanoclay, calcium alginate, and their combinations on estimated green leaf area per plant (means ± 95% confidence intervals) of wheat under well-watered and drought conditions.

Estimated green leaf area per plant i.e. canopy proxy provided the clearest separation among treatments and revealed canopy maintenance as the most drought-sensitive and amendment-responsive growth-level indicator. The drought control exhibited a large decline in canopy proxy (68.7%), consistent with drought-induced restriction of leaf expansion and/or accelerated canopy senescence (**Figure 1**). Amendments substantially attenuated these canopy losses. The strongest canopy protection was again observed for CN (0.5%), which showed only a 7.8% decline from well-watered condition to drought and a 357.3% increase relative to the stressed control under drought. Composite formulations consistently improved canopy proxy under drought relative to the stressed control. These patterns indicate that amendment-mediated mitigation was most strongly expressed through preservation of green canopy area, with secondary effects on shoot fresh weight.

These treatment-dependent trends were statistically supported by the factorial analysis. Type II ANOVA showed significant main effects of Water and Group for shoot fresh weight, canopy proxy, and shoot length, and significant Water × Group interactions for all three traits (P ≤ 0.0159; **Table 2**), confirming that amendment effects differed among formulations and were expressed most strongly under drought.

**Table 2:** Summary of Type II ANOVA results for growth-related traits of wheat in response to water regime (well-watered vs. drought), treatment group, and their interaction (df, degrees of freedom).

Traits	df	F	p-value
Shoot fresh weight			
Water	1	86.392	<0.0001
Group	10	52.891	<0.0001
Water × Group	10	3.054	0.0036
Estimated green leaf area (proxy)			
Water	1	115.138	<0.0001
Group	10	25.512	<0.0001
Water × Group	10	2.274	0.0159
Shoot length			
Water	1	100.054	<0.0001
Group	10	14.982	<0.0001
Water × Group	10	4.26	<0.0001

**Figure 2:**
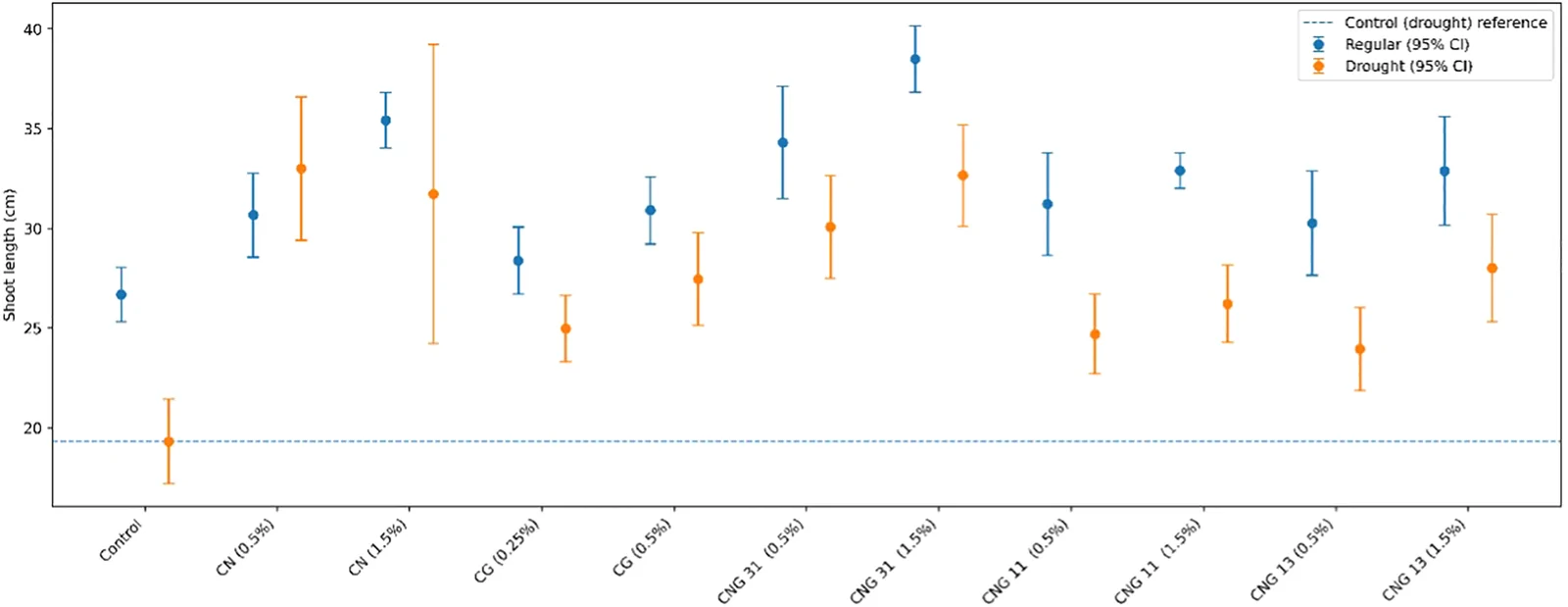
Effects of nanoclay, calcium alginate, and their combinations on shoot length (means ± 95% confidence intervals) of wheat under well-watered and drought conditions.

Shoot length responded in the same direction but with comparatively lower plasticity (**Figure 2**). In the control, drought reduced shoot length by 22.0% (**Supplementary Table 4**). CN (0.5%) maintained higher shoot length under drought (33.0 cm) than the control (19.31 cm). However, proportional changes in shoot length across treatments were consistently smaller than those observed for canopy proxy and shoot fresh weight indicating that structural elongation was a less sensitive indicator of drought mitigation than functional canopy maintenance.

### 3.2 Gas exchange and water-use efficiency

**Figure 3:**
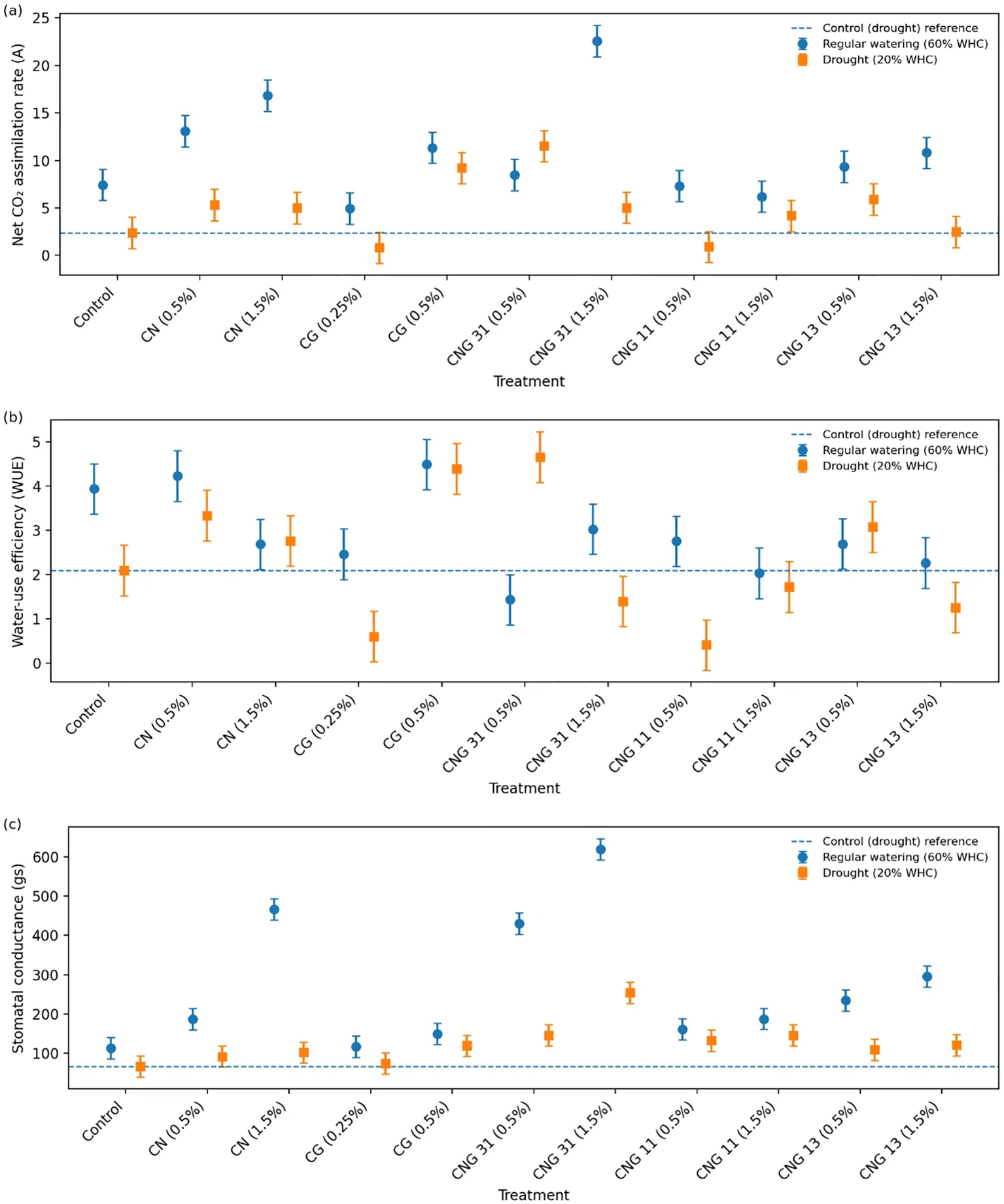
Amendment effects on wheat leaf gas exchange under well-watered and drought conditions. **(A)** Net CO_2_ assimilation rate **(A, B)** instantaneous water-use efficiency (WUE = A/E), and **(C)** stomatal conductance (g_s_) of wheat grown with nanoclay (CN), calcium alginate (CG), and CN–CG composites (CNG 31, CNG 11, CNG 13) under well-watered (60% WHC) and drought (20% WHC) conditions. Points show means with error bars (± 95% confidence intervals).

To determine whether CN, CG and CNG combinations improve wheat physiological performance under water limitation, leaf gas exchange and derived water-use efficiency traits under well-watered (60% WHC) and drought (20% WHC) conditions were quantified (**Figure 3**). Net CO_2_ assimilation (A) showed the clearest drought sensitivity in the control, falling by 68% under drought relative to well-watered condition (**Figure 3A**). Several amendments attenuated this decline, but responses depended strongly on formulation and dose. Under drought, CNG 31 (0.5%) and CG (0.5%) maintained the highest A, reaching approximately 4.8-fold and 3.9-fold higher values than the stressed control, respectively. CN treatments provided intermediate protection, whereas CG (0.25%) and CNG 11 (0.5%) remained at or below the drought-stressed control, indicating limited functional benefit at those configurations.

Water-use efficiency (WUE = A/E) also separated treatments strongly under drought (**Figure 3B**). In the control, WUE declined under drought, whereas CNG 31 (0.5%) and CG (0.5%) showed the highest drought WUE, reaching approximately 2.2-fold and 2.1-fold above the stressed control. Importantly, these higher WUE values coincided with sustained A rather than simply reduced transpiration, indicating improved carbon gain per unit water loss under drought. Variation among treatments was more evident under drought than under well-watered conditions (60% WHC), consistent with the interaction effects identified in the ANOVA (**Supplementary Table 5**). Stomatal conductance (g_s_) declined under drought in the stressed control, but several formulations maintained higher drought-stage g_s_, with the highest values observed in CNG 31 (1.5%) and consistently elevated values observed in CNG 31 (0.5%) and CNG 11 (0.5–1.5%) (**Figure 3C**). However, elevated g_s_ did not necessarily translate into improved carbon gain: for example, CNG 11 (0.5%) showed relatively high drought g_s_ while exhibiting very low A and low WUE under drought. Similarly, CNG 31 (1.5%) maintained the highest g_s_ under drought but only moderate A and low WUE compared with the best-performing treatments.

### 3.3 Photochemistry, spectral indices, and reflectance-derived chlorophyll estimates

Drought reduced PSII efficiency in the untreated control, with F_v_/F_m_ declining from 0.815 (R) to 0.753 (S). Across treatments, drought-stage differences in F_v_/F_m_ were relatively small, but several amendments maintained consistently higher values than the stressed control. Under drought, the best-performing treatments clustered around 0.79–0.80, most notably CNG 31 (1.5%) and CG (0.5%), corresponding to approximately 4–6% higher F_v_/F_m_ than the stressed control. Treatment separation was clearer for ΦPSII under drought. The highest drought-stage ΦPSII occurred in CG (0.5%), which was 11% higher than the stressed control.

Drought strongly altered canopy optical indices in the untreated control. NDVI declined by 18% from R to S, and WBI declined from 0.915 under R to 0.730 under S, representing a 20% decrease. Under drought, NDVI was improved in multiple amended treatments relative to the stressed control. The strongest response was observed in CN (1.5%), where drought-stage NDVI was approximately 40% higher than the stressed control. Composite formulations CNG 31 also showed consistent drought-stage improvements, typically maintaining NDVI 10–16% above the stressed control. These reflectance-based indices were interpreted as supporting indicators of canopy optical status, pigment balance, and hydration-related reflectance signals, rather than as direct measurements of biomass, yield, or agronomic productivity.

A similar pattern was evident for WBI. The highest drought-stage WBI occurred in CG (0.5%), which was approximately 26% higher than the stressed control, while several composites and CN (1.5%) produced intermediate increases, supporting partial maintenance of a water-related optical signature under drought. In the control, drought increased SIPI from 0.678 to 0.751 and increased CRI1 from 0.074 to 0.110. Under drought, SIPI showed comparatively modest treatment shifts, whereas CRI1 was consistently lower in amended treatments than in the stressed control, typically 25–30% lower, indicating a dampened drought-associated carotenoid reflectance response under amendment application.

Reflectance-derived total chlorophyll estimates showed the strongest drought contrast. In the control, reflectance-derived total chlorophyll estimates declined from 20.8 under R to 5.16 under S, representing a 75% reduction. Amendments markedly attenuated chlorophyll depletion under drought. The strongest chlorophyll retention occurred in CG (0.25%), reaching 3.5-fold higher values than the stressed control, followed by CNG 11 (1.5%) and CG (0.5%). Several other formulations, including CN and CNG 31, also sustained higher chlorophyll estimates under drought. Notably, the chlorophyll-retention ranking did not perfectly match the drought-stage ranking of A and WUE, indicating that chlorophyll preservation alone does not fully predict carbon-gain performance under drought.

### 3.4 Cumulative recorded water addition required to maintain target WHC

Cumulative recorded water addition differed significantly between water regimes, among treatment groups, and for the water regime × treatment group interaction. As expected, pots maintained at 60% WHC required greater water addition than pots maintained at 20% WHC (**Table 3**). Under well-watered conditions, the untreated control required 225.7 g pot^-1^ across the recorded weighing dates. CNG 31 (0.5%) and CNG 31 (1.5%) required 196.3 and 171.4 g pot^-1^, corresponding to reductions of 13.0% and 24.1%, respectively, relative to the well-watered control. Under drought, the untreated control required 106.0 g pot^-1^, whereas CNG 31 (0.5%) and CNG 31 (1.5%) required 81.1 and 61.7 g pot^-1^, corresponding to reductions of 23.5% and 41.8%, respectively, relative to the drought-stressed control. Two-factor ANOVA confirmed significant effects of water regime (F = 2072.63, P < 0.0001), treatment group (F = 14.58, P < 0.0001), and their interaction (F = 9.67, P < 0.0001) on cumulative recorded water addition. These values represent water added to restore pots to target WHC and should not be interpreted as direct measurements of plant transpiration, evaporation, total evapotranspiration, field-scale crop water productivity, or yield response.

**Table 3:** Cumulative recorded water addition required to restore pots to target WHC across recorded weighing dates.

Treatment	Well-watered, g pot-1	Reduction vs well-watered control	Drought, g pot-1	Reduction vs drought control
Control	225.7 ± 32.2	—	106.0 ± 8.7	—
CN (0.5%)	243.1 ± 23.7	No reduction	88.2 ± 6.1	16.8%
CN (1.5%)	184.9 ± 25.1	18.1%	80.2 ± 16.0	24.4%
CG (0.25%)	255.1 ± 12.9	No reduction	74.7 ± 30.9	29.5%
CG (0.5%)	253.2 ± 24.8	No reduction	97.8 ± 8.3	7.8%
CNG 31 (0.5%)	196.3 ± 19.3	13.0%	81.1 ± 11.5	23.5%
CNG 31 (1.5%)	171.4 ± 32.2	24.1%	61.7 ± 11.5	41.8%
CNG 11 (0.5%)	279.3 ± 14.1	No reduction	82.0 ± 23.8	22.7%
CNG 11 (1.5%)	224.4 ± 35.3	0.5%	91.3 ± 17.5	13.9%
CNG 13 (0.5%)	254.7 ± 24.2	No reduction	74.6 ± 17.9	29.7%
CNG 13 (1.5%)	272.3 ± 15.5	No reduction	88.5 ± 19.9	16.6%

Values are means ± approximate 95% confidence intervals, n = 5 pots per treatment-water combination. Reductions were calculated relative to the corresponding untreated control within each water regime.

## 4 Discussion

### 4.1 Growth and canopy responses: amendment-mediated buffering of drought impacts

The growth responses indicate that CN, CG, and CNG composite formulations reduced drought-associated constraints on wheat performance, although the magnitude of benefit varied among traits and formulations. This pattern aligns with cereal drought physiology, where declining soil water availability is typically expressed early as reduced leaf expansion and accelerated senescence, leading to reduced canopy development and, in many cases, lower biomass accumulation (Tardieu et al., 2014). In the present study, this growth component was assessed using shoot fresh weight rather than dry biomass.

Consistent with this framework, canopy-related traits (green leaf area proxy/greenness) were among the most responsive indicators of mitigation in this dataset, reflecting their sensitivity to hydraulic limitation and turgor-driven cell expansion processes (Pantin et al., 2011; Tardieu et al., 2014). Canopy metrics should be interpreted as functional integrators rather than direct substitutes for yield; however, their responsiveness under drought makes them useful screening endpoints for amendment performance in water-limited systems (Lopes and Reynolds, 2012).

The observed mitigation is consistent with root-zone buffering mechanisms that moderate declines in plant-available water during soil drying. Alginate-based hydrogels function as localized water reservoirs, temporarily increasing soil water retention and releasing stored moisture as matric potential declines (Hurtado et al., 2022). Nanoclays enhance soil physical properties by improving aggregation, modifying pore-size distribution, and increasing cation exchange capacity, thereby influencing both water retention and nutrient availability (Manjaiah et al., 2019; Abd-Elsalam et al., 2024). In composite systems, clay particles can reinforce polymer networks while polymer matrices improve nanoclay dispersion, potentially stabilizing soil hydraulic function and moderating percolation losses under deficit irrigation (Sarkar et al., 2014; Kong et al., 2019). However, alginate biodegradation and nanoclay aggregation may reduce long-term effectiveness, particularly in coarse-textured soils and repeated wet–dry cycles (Dorochesi et al., 2023; Gupta et al., 2025; Uddin et al., 2024). These interacting processes underscore the importance of formulation optimization for sustained soil–water regulation under drought conditions.

Shoot length exhibited a more conservative response than canopy and shoot fresh-weight traits. This is consistent with evidence that different growth components are regulated by partially independent developmental and hydraulic controls, producing distinct drought sensitivities (Tardieu et al., 2014; Tricker et al., 2018). Agronomically, the pattern suggests that improved performance reflects functional buffering (maintenance of green canopy area and associated shoot fresh-weight support) rather than unrealistic shifts in developmental trajectory, which is more likely to translate into stable performance under deficit irrigation strategies (Condon et al., 2004; Fereres and Soriano, 2007).

### 4.2 Gas exchange and instantaneous water-use efficiency

Leaf gas-exchange results indicate that amendments helped preserve net CO_2_ assimilation (A) under drought, consistent with moderated physiological limitation when root-zone water availability is buffered. Stomatal conductance (g_s_) decreased strongly under drought across treatments, and treatment differentiation in g_s_ was comparatively modest—an expected response because stomatal closure is typically a rapid and dominant adjustment to declining soil water availability. Importantly, drought-induced reductions in A are not governed solely by stomatal limitation; non-stomatal constraints (including mesophyll conductance and biochemical limitations) increasingly contribute as stress progresses (Keenan et al., 2010). Therefore, the clearer treatment separation in A relative to g_s_ suggests that amendments improved overall carbon assimilation under drought, even when stomatal regulation remained strongly water driven.

Under mild-to-moderate drought, WUE often increases because transpiration (E) declines more steeply than assimilation, shifting the balance toward higher A/E (Flexas and Medrano, 2002). However, WUE improvement is agronomically meaningful only when carbon gain is maintained rather than achieved primarily through severe restriction that collapses assimilation (Condon et al., 2004). The concurrent pattern of higher A together with elevated A/E in amended treatments supports a buffering interpretation: amendments likely reduced the intensity of drought limitation, enabling continued assimilation while moderating water loss.

It is important to distinguish instantaneous leaf-level WUE (A/E) from agronomic water productivity, defined as biomass or grain yield per unit of seasonal water supplied. Conceptually, the effective use of water, rather than maximizing instantaneous efficiency alone, is central to crop improvement under drought conditions (Blum, 2009). The present results provide a mechanistic physiological basis for testing potential water-productivity benefits, but they do not directly quantify plant water consumption, seasonal evapotranspiration, whole-season biomass production, or grain yield. Field-scale validation under deficit irrigation is therefore required before inferring agronomic water-productivity gains (Rockström et al., 2010).

### 4.3 Photochemistry

Chlorophyll fluorescence results showed significant drought effects on PSII performance (F_v_/F_m_ and ΦPSII), whereas treatment modulation was generally weaker and less consistent than for gas exchange. This hierarchy is physiologically plausible. Under moderate drought, photosynthesis is often constrained primarily by diffusive and metabolic limitations, while substantial declines in F_v_/F_m_ typically appear under more severe or prolonged stress and reflect sustained photoinhibition or chronic damage (Baker, 2008). Wheat can maintain PSII integrity through photoprotective mechanisms under moderate drought, with stronger impairment emerging as stress intensifies (Zivcak et al., 2013; Abdullaev et al., 2024). Accordingly, fluorescence traits are best framed as supportive indicators of photochemical status that complement gas-exchange responses rather than serving as the primary basis for treatment ranking (Maxwell and Johnson, 2000).

Within fluorescence metrics, a clearer response in ΦPSII than in F_v_/F_m_ is expected because ΦPSII reflects operational efficiency under illumination and responds to regulatory changes in energy use and electron transport, whereas F_v_/F_m_ is more diagnostic of sustained impairment after dark adaptation (Maxwell and Johnson, 2000; Baker, 2008). Thus, the fluorescence pattern is consistent with amendment-related modulation of dynamic photochemical regulation under drought without large differences in chronic PSII damage.

### 4.4 Spectral indices and pigments

Spectral indices (NDVI, WBI, SIPI) and pigment traits showed strong drought sensitivity with selective treatment responsiveness, supporting their value as complementary indicators of canopy condition. NDVI is widely used as a proxy for green canopy status and photosynthetically active biomass, but it can saturate at higher canopy density and is influenced by canopy structure and measurement geometry; therefore, it is most informative when interpreted alongside direct physiological measurements (Jiang et al., 2006). WBI is linked to vegetation water status through water-related absorption features, and SIPI reflects shifts in pigment balance under stress, providing additional context for drought-driven changes (Claudio et al., 2006). The observation that several treatments increased NDVI and/or WBI relative to the stressed control is consistent with partial maintenance of canopy optical condition and hydration-related reflectance signals; however, these indices should be interpreted as supporting evidence and not as direct measures of productivity, biomass accumulation, or yield.

Reflectance-derived chlorophyll estimates displayed strong drought sensitivity, with chlorophyll depletion in the stressed control and partial mitigation by several amendments. Nevertheless, pigment retention and assimilation did not necessarily align perfectly across formulations, indicating that chlorophyll preservation should be interpreted primarily as maintenance of pigment pool integrity and potential photosynthetic capacity. Net CO_2_ assimilation remains additionally constrained by stomatal and non-stomatal limitations that can persist even when pigments are partially protected (Flexas and Medrano, 2002). Finally, the limited responsiveness of CRI1 is consistent with its dependence on carotenoid pool adjustments, which may be less variable among treatments in relatively short or moderate drought scenarios where dominant signals relate to canopy hydration and functional area rather than major carotenoid restructuring (Gitelson et al., 2002).

### 4.5 Agronomic relevance, study limitations, and future research priorities

In water-limited production systems, the partial maintenance of canopy function and net carbon assimilation observed at 20% WHC is directly relevant to deficit irrigation strategies designed to improve water productivity (biomass or yield per unit water supplied) rather than maximizing production per unit land area (Passioura, 1977; Fereres and Soriano, 2007; Geerts and Raes, 2009). In practical terms, treatment-associated preservation of green canopy function and net carbon assimilation under water deficit provides a physiologically meaningful basis for stabilizing crop performance in arid and semi-arid systems, where canopy persistence and continued CO_2_ uptake underpin biomass formation and, ultimately, yield resilience (Condon et al., 2004; Hatfield and Dold, 2019). Nonetheless, the present conclusions are confined to growth and physiological endpoints; direct inference on grain yield response and field-scale water productivity requires further validation.

Several study limitations should be considered when interpreting these findings. First, the pot-based design imposes restricted rooting volume and simplified hydraulic gradients compared with field soils, which may influence root exploration patterns, soil–root contact, and amendment distribution within the bulk soil matrix. These constraints may modify drought progression dynamics and root-zone buffering relative to field conditions. Second, the experimental duration did not resolve persistence of amendment effects under repeated wet–dry cycles or across seasons, an important consideration for biodegradable biopolymers and composite stability (Malik et al., 2022). In addition, although cumulative recorded water addition required to restore pots to target WHC was calculated, evaporation and transpiration were not partitioned separately. Consequently, the recorded water-addition values should not be interpreted as direct plant water consumption, total evapotranspiration, or whole-season crop water productivity. Direct inference on agronomic water productivity requires field validation with seasonal evapotranspiration, dry biomass, grain yield, and standardized deficit-irrigation metrics. Third, shoot dry biomass, root biomass, total plant dry biomass, and root-to-shoot ratio were not quantified. Therefore, shoot fresh weight should be interpreted as a short-term vegetative growth indicator rather than as a substitute for dry biomass, final biomass production, or yield. Root system traits and rhizosphere processes were also not quantified, limiting mechanistic attribution of treatment effects to pathways such as altered root exploration, rhizosphere hydraulic properties, or nutrient acquisition under water deficit. Finally, while spectral indices provide complementary canopy-scale proxies, they are sensitive to measurement geometry and scale, constraining extrapolation from pot-scale measurements to canopy-scale remote sensing applications (Jiang et al., 2006).

From a practical perspective, economic feasibility, amendment longevity, and scalability under commercial irrigation systems must be evaluated. Cost–benefit analyses incorporating amendment persistence, potential yield stabilization, and independently measured irrigation or evapotranspiration responses are necessary to determine adoption potential in water-limited agricultural systems. Future research should prioritize (i) field trials across contrasting soil textures and irrigation regimes to test robustness under agronomically realistic conditions, (ii) multi-cycle and multi-season assessments to quantify functional longevity and stability under repeated drying–rewetting, (iii) integration of root architecture and rhizosphere measurements (e.g., root length density, hydraulic conductance, soil–root contact, nutrient dynamics) to strengthen mechanistic inference, and (iv) explicit linkage of physiological buffering to grain yield and whole-season water productivity outcomes using standardized deficit-irrigation metrics.

## 5 Conclusions

Nanoclay, calcium alginate, and CN–CG composites attenuated drought effects on wheat vegetative growth and leaf physiological performance under controlled greenhouse conditions. Under 20% WHC, amendments improved canopy maintenance and shoot fresh weight relative to the untreated stressed control, with canopy-related and reflectance-based traits providing sensitive supporting indicators of drought buffering rather than direct measures of productivity or yield. Treatment performance was trait-specific: CN (0.5%) showed the strongest mitigation of drought effects on growth-related traits, whereas CNG 31 (0.5%) showed the strongest drought-stage gas-exchange response, with net CO_2_ assimilation and instantaneous leaf-level WUE exceeding the stressed control.

Based on recorded pot-mass restoration data, selected composite formulations reduced the water addition required to maintain target WHC. CNG 31 (0.5%) reduced recorded water addition by approximately 13.0% under well-watered conditions and 23.5% under drought, whereas CNG 31 (1.5%) showed the largest reduction, 24.1% under well-watered conditions and 41.8% under drought. These values represent water added to restore pots to target WHC and should not be interpreted as direct measurements of plant water consumption, total evapotranspiration, grain yield response, or field-scale crop water productivity.

Overall, the findings identify CN, CG, and CN–CG composites as promising soil amendment candidates for mitigating short-term vegetative and physiological drought responses in wheat. Field validation is required to determine whether these greenhouse responses translate into improved root development, dry biomass accumulation, grain yield, seasonal water productivity, amendment persistence, and cost-effective performance under agronomically realistic deficit-irrigation conditions.

## Data Availability

The original contributions presented in the study are included in the article/**Supplementary Material**. Further inquiries can be directed to the corresponding authors.
