# Supplementary material for: Nanoclay, calcium alginate, and composite soil amendments mitigate drought effects on wheat growth and water-use efficiency
Source: Front Plant Sci. 2026 Jul 15;17:1846027. doi: 10.3389/fpls.2026.1846027 (PMC13414955; doi:10.3389/fpls.2026.1846027)
Supplement: Supplementary file 1 [file Table1.docx]

**Supplementary file**

**Table S1**: Treatment groups

| **Treatment Code** | **Description** | **CN %** | **CG %** |
| --- | --- | --- | --- |
| CG | Calcium alginate only | – | – |
| CN | Clay nanoparticles only | – | – |
| CNG11 | CN–CG combination (equal CN and CG) | 25.0 | 25.0 |
| CNG13 | CN–CG combination (low CN, high CG) | 12.5 | 37.5 |
| CNG31 | CN–CG combination (high CN, low CG) | 37.5 | 12.5 |

**Table 2.** DLS hydrodynamic particle size and polydispersity index of CN, CG and composite formulations.

| Parameters | CN | CG | CNG31 | CNG11 | CNG13 |
| --- | --- | --- | --- | --- | --- |
| DLS particle size (nm) | 500 | 841 | 548 | 584 | 714 |
| DLS polydispersity index | 0.6325 | 0.1601 | 0.5432 | 0.3231 | 0.2112 |

**Table S3**. Effects of nanoclay, calcium alginate, and their combinations on shoot length (means ± 95% confidence intervals) of wheat under regular and drought conditions.

| Treatment | Dose (%) | Water regime | Mean | 95% CI |
| --- | --- | --- | --- | --- |
| Control | – | Regular | 26.68 | 24.28 – 29.08 |
|  | – | Drought | 19.31 | 16.91 – 21.71 |
| CN | 0.5 | Regular | 30.67 | 28.27 – 33.07 |
|  | 0.5 | Drought | 33.0 | 30.60 – 35.40 |
|  | 1.5 | Regular | 35.41 | 33.01 – 37.81 |
|  | 1.5 | Drought | 21.72 | 18.50 – 24.94 |
| CG | 0.25 | Regular | 28.38 | 25.98 – 30.78 |
|  | 0.25 | Drought | 24.97 | 22.57 – 27.37 |
|  | 0.5 | Regular | 30.9 | 28.50 – 33.30 |
|  | 0.5 | Drought | 27.46 | 25.06 – 29.86 |
| CNG 31 | 0.5 | Regular | 34.3 | 31.90 – 36.70 |
|  | 0.5 | Drought | 30.08 | 27.68 – 32.48 |
|  | 1.5 | Regular | 38.49 | 36.09 – 40.89 |
|  | 1.5 | Drought | 32.65 | 30.38 – 34.93 |
| CNG 11 | 0.5 | Regular | 31.23 | 28.83 – 33.63 |
|  | 0.5 | Drought | 24.7 | 22.30 – 27.10 |
|  | 1.5 | Regular | 32.9 | 30.50 – 35.30 |
|  | 1.5 | Drought | 26.21 | 23.81 – 28.61 |
| CNG 13 | 0.5 | Regular | 30.26 | 27.86 – 32.66 |
|  | 0.5 | Drought | 23.95 | 21.55 – 26.35 |
|  | 1.5 | Regular | 32.88 | 30.48 – 35.28 |
|  | 1.5 | Drought | 28.01 | 25.61 – 30.41 |

**Table S4.** Percentage changes in growth-related traits of wheat under drought relative to regular watering and stressed control. They were calculated from model-estimated means. R → S indicates percentage change from regular watering (60% WHC) to drought (20% WHC) within each treatment. Percentage change vs Control-S indicates relative performance under drought compared with the untreated stressed control. Values reflect relative changes from low baseline values under drought and should be interpreted as comparative indicators rather than absolute magnitudes

| Trait /Treatment group | % change (R → S) | % change vs Control-S |  |
| --- | --- | --- | --- |
| Shoot fresh weight | | | |
| Control | -35.5 | 0.0 |  |
| CN (0.5%) | -12.0 | 121.9 |  |
| CN (1.5%) | -38.8 | 27.9 |  |
| CG (0.25%) | -37.1 | 35.7 |  |
| CG (0.5%) | -12.7 | 69.1 |  |
| CNG 31 (0.5%) | -40.7 | 12.5 |  |
| CNG 31 (1.5%) | -34.2 | 29.3 |  |
| CNG 11 (0.5%) | -25.8 | 42.2 |  |
| CNG 11 (1.5%) | -25.3 | 55.7 |  |
| CNG 13 (0.5%) | -19.4 | 24.0 |  |
| CNG 13 (1.5%) | -22.9 | 45.0 |  |
| Estimated green leaf area (proxy) | | | |
| Control | -68.7 | 0.0 |  |
| CN (0.5%) | -7.8 | 357.3 |  |
| CN (1.5%) | -40.0 | 70.9 |  |
| CG (0.25%) | -39.8 | 121.9 |  |
| CG (0.5%) | -14.3 | 264.4 |  |
| CNG 31 (0.5%) | -45.2 | 148.3 |  |
| CNG 31 (1.5%) | -41.7 | 212.8 |  |
| CNG 11 (0.5%) | -27.1 | 150.6 |  |
| CNG 11 (1.5%) | -26.3 | 175.2 |  |
| CNG 13 (0.5%) | -20.6 | 189.7 |  |
| CNG 13 (1.5%) | -23.8 | 204.1 |  |
| Shoot length | | | |
| Control | -22.0 | 0.0 |  |
| CN (0.5%) | -6.5 | 45.0 |  |
| CN (1.5%) | -18.9 | 24.0 |  |
| CG (0.25%) | -17.4 | 29.3 |  |
| CG (0.5%) | -8.1 | 69.1 |  |
| CNG 31 (0.5%) | -19.8 | 27.9 |  |
| CNG 31 (1.5%) | -16.3 | 35.7 |  |
| CNG 11 (0.5%) | -13.4 | 42.2 |  |
| CNG 11 (1.5%) | -11.2 | 55.7 |  |
| CNG 13 (0.5%) | -9.0 | 25.0 |  |
| CNG 13 (1.5%) | -10.5 | 33.0 |  |

**Table S5.** Summary of Type II ANOVA results for gas exchange, photosystem performance, spectral indices, and pigment-related traits of wheat in response to water regime (regular vs. drought), treatment group, and their interaction (*df*, degrees of freedom).

| Trait | Source | df | F | p-value |
| --- | --- | --- | --- | --- |
| Net CO₂ assimilation rate (A) | Water | 1 | 285.972 | <0.0001 |
|  | Group | 10 | 32.97 | <0.0001 |
|  | Water × Group | 10 | 21.858 | <0.0001 |
| Stomatal conductance (gs) | Water | 1 | 628.58 | <0.0001 |
|  | Group | 10 | 114.951 | <0.0001 |
|  | Water × Group | 10 | 47.912 | <0.0001 |
| Transpiration rate (E) | Water | 1 | 988.573 | <0.0001 |
|  | Group | 10 | 146.967 | <0.0001 |
|  | Water × Group | 10 | 60.53 | <0.0001 |
| Water-use efficiency (WUE) | Water | 1 | 21.973 | <0.0001 |
|  | Group | 10 | 21.379 | <0.0001 |
|  | Water × Group | 10 | 14.469 | <0.0001 |
| Maximum quantum efficiency of PSII (Fv/Fm) | Water | 1 | 131.38 | <0.0001 |
|  | Group | 10 | 3.629 | 0.0003 |
|  | Water × Group | 10 | 2.051 | 0.0339 |
| Effective quantum yield of PSII (ΦPSII) | Water | 1 | 275.116 | <0.0001 |
|  | Group | 10 | 10.141 | <0.0001 |
|  | Water × Group | 10 | 3.779 | 0.0002 |
| Normalized Difference Vegetation Index | Water | 1 | 10.124 | 0.002 |
| (NDVI) | Group | 10 | 3.722 | 0.0003 |
|  | Water × Group | 10 | 2.337 | 0.0163 |
| Water Band Index (WBI) | Water | 1 | 37.075 | <0.0001 |
|  | Group | 10 | 3.437 | 0.0007 |
|  | Water × Group | 10 | 2.228 | 0.0221 |
| Structure Insensitive Pigment Index (SIPI) | Water | 1 | 45.493 | <0.0001 |
|  | Group | 10 | 3.709 | 0.0003 |
|  | Water × Group | 10 | 2.383 | 0.0144 |
| Carotenoid Reflectance Index 1 (CRI1) | Water | 1 | 4.685 | 0.0329 |
|  | Group | 10 | 1.151 | 0.3339 |
|  | Water × Group | 10 | 0.421 | 0.9332 |
| Chlorophyll a | Water | 1 | 375.953 | <0.0001 |
|  | Group | 10 | 8.491 | <0.0001 |
|  | Water × Group | 10 | 5.4 | <0.0001 |
| Chlorophyll b | Water | 1 | 434.887 | <0.0001 |
|  | Group | 10 | 4.285 | <0.0001 |
|  | Water × Group | 10 | 5.482 | <0.0001 |
| Total chlorophyll (a + b) | Water | 1 | 524.859 | <0.0001 |
|  | Group | 10 | 5.696 | <0.0001 |
|  | Water × Group | 10 | 6.333 | <0.0001 |
